# Variations in Guidelines for Diagnosis of Child Physical Abuse in High-Income Countries: A Systematic Review

**DOI:** 10.1001/jamanetworkopen.2021.29068

**Published:** 2021-11-17

**Authors:** Flora Blangis, Slimane Allali, Jérémie F. Cohen, Nathalie Vabres, Catherine Adamsbaum, Caroline Rey-Salmon, Andreas Werner, Yacine Refes, Pauline Adnot, Christèle Gras-Le Guen, Elise Launay, Martin Chalumeau, and the European Confederation of Primary Care Paediatricians (ECPCP) research group

**Affiliations:** 1 Obstetrical, Perinatal and Pediatric Epidemiology Research team, Centre of Research in Epidemiology and Statistics, Université de Paris, INSERM, F-75004, Paris, France; 2 Inserm CIC 1413, Nantes University Hospital, F-44000, Nantes, France; 3 Department of General Pediatrics and Pediatric Infectious Diseases, AP-HP, Necker-Enfants Malades Hospital, Université de Paris, F-75015, Paris, France; 4 Unité d’Accueil des Enfants en Danger, Nantes University Hospital, F-44000, Nantes, France; 5 Department of Pediatric Radiology, AP-HP, Bicêtre Hospital, F-94270, Le Kremlin Bicêtre, France; 6 Pediatrics and Forensic Unit, AP-HP, Hôtel-Dieu Hospital, F-75004, Paris, France; 7 AFPA, Association Française de Pédiatrie Ambulatoire, F-30400, Villeneuve les Avignons, France; 8 Department of Pediatric Emergency Care, Nantes University Hospital, F-44000, Nantes, France

## Abstract

**Importance:**

The highly variable practices observed regarding the early detection and diagnostic workup of suspected child physical abuse contribute to suboptimal care and could be partially related to discrepancies in clinical guidelines.

**Objective:**

To systematically evaluate the completeness, clarity, and consistency of guidelines for child physical abuse in high-income countries.

**Evidence Review:**

For this systematic review, national or regional guidelines that were disseminated from 2010 to 2020 related to the early detection and diagnostic workup of child physical abuse in infants aged 2 years or younger by academic societies or health agencies in high-income countries were retrieved. The definitions of sentinel injuries and the recommended diagnostic workup (imaging and laboratory tests) for child physical abuse were compared. Data were analyzed from July 2020 to February 2021.

**Findings:**

Within the 20 included guidelines issued in 15 countries, 168 of 408 expected statements (41%) were missing and 10 statements (4%) were unclear. Among 16 guidelines characterizing sentinel injuries, all of them included skin injuries, such as bruises, hematoma, or burns, but only 8 guidelines (50%) included intraoral injuries and fractures. All 20 guidelines agreed on the indication for radiological skeletal survey, head computed tomography, and head magnetic resonance imaging but differed for those of bone scintigraphy, follow-up skeletal survey, spinal magnetic resonance imaging, cranial ultrasonography, chest computed tomography, and abdominal ultrasonography and computed tomography. Additionally, 16 guidelines agreed on exploring primary hemostasis and coagulation but not on the tests to perform, and 8 guidelines (50%) mentioned the need to investigate bone metabolism.

**Conclusions and Relevance:**

These findings suggest that guidelines for the diagnosis of child physical abuse in infants were often clear but lacked completeness and were discrepant on major issues. These results may help identify priorities for well-designed original diagnostic accuracy studies, systematic reviews, or an international consensus process to produce clear and standardized guidelines to optimize practices and infant outcomes.

## Key Points

**Question:**

Are clinical guidelines for the early detection and diagnostic workup of child physical abuse complete, clear, and consistent across high-income countries?

**Findings:**

In this systematic review that included 20 clinical guidelines issued in 15 countries, guidelines were clear but incomplete and discrepant, particularly in the definition of sentinel injuries and in recommendations for exploratory laboratory testing and advanced imaging.

**Meaning:**

This systematic review found a lack of standardized guidelines for the identification and management of child physical abuse, which may contribute to practice variation.

## Teaser

This systematic review evaluates the completeness, clarity, and consistency of guidelines from high-income countries for the detection and diagnosis of child physical abuse.

## Introduction

Physical abuse is estimated to occur in 4% to 16% of the population younger than 18 years in high-income countries,^1^ and the World Health Organization has considered child physical abuse (CPA) an international priority and has developed a vast program of CPA prevention.^2^ CPA is more frequent in infants 2 years or younger in whom, in the absence of criteria of certainty other than the rare confession of the perpetrator, diagnosis is complex and relies on a combination of social and clinical evaluations, imaging, and laboratory tests.^3,4,5^ False-negative results expose infants to a risk of recurrence estimated at 35% to 50%,^6,7^ with its associated morbidity and mortality in the short^8,9,10^ and long term.^11,12^ However, false-positive results may delay the diagnosis of severe underlying diseases, such as bone fragility (eg, osteogenesis imperfecta) or bleeding disorders (eg, hemophilia) and lead to an inappropriate child protection decision.^13^ Thus, early detection of CPA based on sentinel injuries (ie, injuries in noncruising infants or with implausible explanations),^14^ alone or interpreted with the help of clinical decision rules, such as the TEN-4 rule (ie, bruises on the torso, ears, and neck in children younger than 4 years may be indicative of CPA),^15^ and accurate diagnostic workup with imaging and laboratory tests are of paramount importance.

To help physicians optimize the detection and diagnosis of CPA and consider differential diagnoses, clinical guidelines have been developed by academic societies and health agencies. Despite these efforts at standardization, several studies reported suboptimal practices by health care practitioners. For example, in 2018, 36% of physicians in 4 European countries considered that an infant aged 10 weeks with bleeding from the mouth was not a child protection concern.^16^ In a French national survey performed in 2015, only 28% of pediatricians would prescribe magnetic resonance imaging (MRI) of the head for the diagnostic workup of CPA in an infant aged 9 months with a fractured femur, numerous bruises, and head trauma.^17^ Lack of completeness, clarity, and consistency are among the reasons why clinical guidelines fail to standardize practices and thus mislead health professional practices.^18,19^ For example, we recently identified a between-guideline discrepancy for the imaging workup to be performed to detect skeletal injuries when CPA is suspected, notably the role of bone scintigraphy.^20,21^ Identifying the specific fields for which guidelines lack completeness, clarity, or consistency for the early detection and diagnostic workup of CPA could help prioritize clinical questions requiring original diagnostic studies, systematic reviews (as performed in the aforementioned example of bone scintigraphy),^20^ or an international consensus process. Our objective was to systematically investigate the completeness, clarity, and consistency of clinical guidelines for the early detection and diagnostic workup of CPA that were issued by academic societies and health agencies in high-income countries.

## Methods

This systematic review was performed according to guidance from the Centre for Reviews and Dissemination^22^ and its reporting followed the Preferred Reporting Items for Systematic Reviews and Meta-analyses (PRISMA) reporting guideline. A protocol was registered (Prospero No. CRD42020203809). In this systematic review, we aimed to identify, analyze, and compare all guidelines that were issued after 2010 by academic societies or health agencies in high-income countries with a guidance on the early detection and/or diagnostic workup of CPA in infants aged 2 years or younger.^23^ This age limit was selected because most nonclinically visible injuries are found before this age.^24,25^ The database searches and data extraction and synthesis were performed by 2 of us (F.B. and Y.R.) independently. Disagreements were resolved by consensus or by consulting 1 or several other review authors (F.B., S.A., Y.R., and M.C.). We translated the non-English guidelines with the help of native-speaker physicians.

### Search Strategy and Selection Criteria

We searched MEDLINE (via PubMed), Web of Science, Google Scholar, websites reporting guidelines (eg, SUMSearch 2,^26^ Guidelines International Network,^27^ and Trip Database^28^) from inception to June 15, 2020 (last update), with no language restrictions, as well as the websites of academic societies and health agencies in the 24 countries with the highest incomes (eTable 1 in Supplement 1).^23^ The search strategy for databases combined groups of keywords pertaining to child abuse, diagnosis, and guidelines (eTable 2 in Supplement 1). We assessed potential guidelines for inclusion by screening titles, abstracts, and, eventually, full texts of all search results. When several guidelines had been published by the same academic society or health agency since 2010, we included only the most recent one. We also contacted the national chairs of the countries covered by the European Confederation of Primary Care Paediatricians (ECPCP) (eTable 1 in Supplement 1) and asked them about current guidelines in their countries. Finally, we screened reference lists of included guidelines. When guidelines were published in several parts,^3^ we considered them as a single guideline. When guidelines endorsed another guideline,^29,30^ we chose the most recent one.^4^

We included all the guidelines aimed at providing general guidance for the early detection and diagnostic workup of CPA. For some specific review questions, we also included guidelines with a narrower scope, notably those regarding the recommended diagnostic workup to detect abusive head trauma or skeletal injuries.

### Data Extraction and Synthesis

For each included guideline, we extracted its characteristics, including country, year of dissemination, development process with the report of the group membership involved, search methods (eg, systematic review, in particular the description of the scope and grading or rating of the recommendations),^31,32,33^ and specific content. We classified the scope of each guideline as a general guidance for the early detection and diagnostic workup of CPA or a narrower one. We compared the presence and the detailed content of a definition of sentinel injuries. We listed the recommended diagnostic workup (imaging and laboratory tests) for CPA.

### Data Analysis

Given the numerous tests proposed, we restricted the subsequent analyses to those suggested in more than 2 guidelines. From the guidelines’ text, we classified whether the test was recommended or not, and whether it was recommended systematically or on a case-by-case basis according to the clinical context or if the recommendation was unclear. We calculated the overall proportion of missing statements and the proportion of unclear statements among the nonmissing statements for all tests.

To compare the guidelines’ contents, we grouped the tests according to the 4 domains they dealt with: detection of skeletal, head and spine, or thoracoabdominal injuries and exploration of differential diagnoses. For each diagnostic test, we calculated the proportion of guidelines providing a statement for it if it was expected given their scope. Analyses were conducted from July 2020 to February 2021.

## Results

### Guidelines Characteristics

We identified 267 records by database searches and 624 records by searching other sources (eFigure in Supplement 1). From 790 unique search results, we identified 20 guidelines,^3,34,35,36,37,38,39,40,41,42^ including 10 in English,^4,5,25,43,44,45,46,47,48,49^ issued by academic societies or health agencies in 15 countries between 2010 and 2020 (eTable 3 in Supplement 1). There were 11 guidelines^3,4,5,25,36,41,42,46,47,48,49^ (55%) that reported their development process, and they were developed by multidisciplinary groups; 5 guidelines^4,41,42,46,47^ reported a literature review, 2 guidelines^5,48^ reported a systematic review, and 4 guidelines^3,25,36,49^ provided a grading or a rating of the level of proof or the strength of recommendations.

Among 20 guidelines identified, 16 guidelines^3,4,34,35,36,37,38,40,41,42,43,44,45,46,47,48^ provided general guidance in case of suspected CPA, including imaging and laboratory tests to be performed; 3 guidelines focused on the diagnostic workup for inflicted skeletal injuries^46^ or abusive head trauma,^3,47^ and 4 guidelines,^5,25,39,49^ all issued by radiology societies, provided guidance for the imaging tests to be performed (eTable 3 in Supplement 1). Thus, given the scope of these 20 guidelines, for 16 guidelines,^3,4,34,35,36,37,38,40,41,42,43,44,45,46,47,48^ we expected a definition of sentinel injuries and the indication of imaging and laboratory tests, including those for the diagnostic workup for differential diagnoses (ie, bone fragility and bleeding disorders). For 4 guidelines,^5,25,39,49^ we expected only the indication for imaging tests (eTable 4 in Supplement 1).

We identified 28 recommended tests (or groups of laboratory tests): 23 tests (82%) were suggested in more than 2 guidelines (eTable 5 in Supplement 1), and 5 tests (18%) were suggested in 2 guidelines or fewer (ie, whole-body MRI, electroencephalography, bone ultrasonography, pelvis computed tomography [CT], new-born screen review). Thus, a total of 408 statements were expected, given the scope of the guidelines (23 statements each for 16 guidelines^3,4,34,35,36,37,38,40,41,42,43,44,45,46,47,48^ and 10 statements each for 4 guidelines^5,25,39,49^). We considered that 168 statements (41%) were missing, and among the nonmissing statements, 10 statements (4%) were unclear (Table 1; eTable 5 in Supplement 1).

**Table 1.** Examples of Interpretations Performed During the Analysis of Guidelines for the Early Detection and/or the Diagnostic Workup of Child Physical Abuse in Infants

Example of wording	Interpretation
**Explanatory sentences**	
“Systematic,” “systematically,” “should,” “is required,” “in all children”	The test has to be performed systematically
“Could,” “may,” “might,” “consider,” “in case of,” “is often used,” “if the child is at risk for,” “possibly,” “based on findings”	The test should be performed on a case-by-case basis, according to the clinical context
“Consider neuroimaging,”^46^ “additional imaging studies may be indicated”^46^	The recommendation is unclear (regarding the neuroimaging and additional imaging to be performed)
**List of tests**	
“First line investigation”	The test has to be performed systematically
“Second line investigation”	The test should be performed on a case-by-case basis according to the clinical context
Tests are listed with no conditional settings	The test has to be performed systematically

### Guidelines Content

#### Sentinel Injury Definition

All 16 expected guidelines^3,4,34,35,36,37,38,40,41,42,43,44,45,46,47,48^ provided a definition of sentinel injuries (Table 2). Six guidelines^34,36,37,38,40,43^ gave a brief characterization of sentinel injuries and focused on only skin injuries: hematoma, bruises, burns, abrasions, lacerations, and scars. Two guidelines^4,47^ added the TEN-4 rule for assessing children with bruises according to the age of the child and the location of the bruises. Ten guidelines^3,4,40,41,42,44,45,46,47,48^ added the skeletal injuries, intraoral injuries, intracranial injuries, or abdominal injuries; 10 guidelines^3,4,35,42,44,45,46,47,48^ gave details on the location of these injuries, 5 guidelines^4,38,42,44,48^ mentioned the number of injuries by terms such as *multiple*, *in cluster*, or *in great quantity*, 2 guidelines^35,42^ gave details on the size, and 6 guidelines^4,35,42,44,45,48^ gave details on the pattern of the injuries. Eight guidelines^3,4,42,44,45,46,47,48^ added injuries in noncruising children. The specific term *sentinel injury* was found in only 3 guidelines.^3,4,47^

**Table 2.** Definition of Sentinel Injuries

Source	Country	Wording^a^	Definition points covered					
			Type	Location	No.	Size	Pattern	Mobility noncruising child
**With the term *sentinel injury***								
Haute Autorité de Santé,^3^ 2017	France	“Sentinel injuries: skin lesions, particularly bruises or hematoma, lesions of the ear-nose-throat sphere, especially inside the mouth, fractures in a noncruising child”	Yes	Yes	No	No	No	Yes
Christian and the Committee on Child Abuse and Neglect, American Academy of Pediatrics,^4^ 2015	United States	“Previous sentinel injuries, defined as inflicted injuries that are minor and recognized by physicians or parents before the recognition that the child has been abused.… The majority of sentinel injuries are bruises, intraoral injuries, including frena tears, or fractures.… Abused children may have clustering of bruises…with handprints or looped marks.… Bruises are notably rare in perambulatory infants.… Bruises to the torso, ears, or neck in children ≤4 years of age are predictive of abuse ‘TEN 4’”	Yes	Yes	Yes	No	Yes	Yes
Narang et al; the Council on Child Abuse and Neglect, American Academy of Pediatrics,^47^ 2020	United States	“…80% of those sentinel injuries were bruises.… Particular attention should be given to ‘TEN-4’ bruising (bruising of the torso, ears, and neck in children younger than 4 y or any bruising in an infant younger than 4 mos). Oral injuries in infants, such as frenulum tears, may also accompany or precede abusive head trauma and should prompt consideration of abuse”	Yes	Yes	No	No	No	Yes
**Without the term *sentinel injury***								
New South Wales Government,^43^ 2014	Australia	“Bruise, abrasion, laceration, burn, scar etc”	Yes	No	No	No	No	No
Government of Western Australia Department of Health,^44^ 2017	Australia	“Any injury/bruising in pre-mobile infants…lacerations and welts burns, including cigarette burns, and scalds, ingestion of poisonous substances, facial, head or neck bruising, multiple injuries or bruises, including bruising and marks that show the shape of the object that caused it e.g. a belt buckle, bruising of the pinna external ear”	Yes	Yes	Yes	No	Yes	Yes
Canadian Paediatric Society,^46^ 2018	Canada	“Bruising or oral trauma, particularly in young infants. Bruises, especially on the child’s trunk, ears and neck, may be a marker for inflicted trauma.… Intracranial and abdominal injuries”	Yes	Yes	No	No	No	Yes
Arbeitsgemeinschaft der Wissenschaftlichen Medizinischen Fachgesellschaften,^36^ 2019	Germany	“Hematoma and thermic injuries”	Yes	No	No	No	No	No
Association of Family Physicians,^35^ 2014	Israel	“Bruises in the neck…bites or beats the size of an adult’s palm, burn with sharp borders, shaped like gloves/socks and a cigarette”	Yes	Yes	No	Yes	Yes	No
Japan Pediatric Society,^34^ 2014	Japan	“Burns, bruises, abrasions, lacerations”	Yes	No	No	No	No	No
Nederlandse Vereniging voor Kindergeneeskunde,^42^ 2016	The Netherlands	“Very young child with serious injuries without direct explanation – Unusual site of injury…burns, fractures, inflicted brain injury…a recognizable pattern of an object or body part…one or more bruises in a premobile child…. Circular burns with deep craters corresponding in size to cigarette burns”	Yes	Yes	Yes	Yes	Yes	Yes
The Paediatric Society of New Zealand,^45^ 2016	New Zealand	“Any infant who has any bruise or fracture and is not yet cruising, climbing or walking. …an apparently trivial bruise to the head of a young infant with no signs of concussion may be a marker of serious risk”	Yes	Yes	No	No	Yes	Yes
Nasjonalt kunnskapassenter om vold og traumatisk stress,^41^ 2018	Norway	“Tears, cuts, wounds, bruises, scars, rashes, hair loss.… Oral cavity / pharynx: mucosal damage, frenulum damage.… Look behind the ear and on the helix for blood clots.… Look at the neck for skin changes”	Yes	Yes	No	No	No	No
Asociación Española de Pediatría and Sociedad Española de Urgencias Pediátricas,^38^ 2010	Spain	“Bruises and burns…, hematomas in covered, in great quantity…, multiple scars”	Yes	No	Yes	No	No	No
Swedish Pediatric Society,^40^ 2019	Sweden	“Bruises…wounds”	Yes	No	No	No	No	No
Swiss Society of Paediatrics,^37^ 2017	Switzerland	“Injuries / hematoma”	Yes	No	No	No	No	No
Royal College of Paediatrics and Child Health,^48^ 2019	United Kingdom	“Bruises…bites…fractures…burns and scalds.… Bruising in children who are not independently mobile.… Multiple bruising or bruises in clusters.… Bruises to the face, eyes, ears, trunk, arms, buttocks and hands.… Bruises that carry the imprint of a hand, ligature or implement used”	Yes	Yes	Yes	No	Yes	Yes

^a^ Text is given verbatim when the original was in English, or translation is given for non-English original.

#### Detection of Skeletal Injuries

All 20 guidelines gave a recommendation regarding which diagnostic tests should be performed to detect skeletal injuries. Radiological skeletal survey was mentioned in all 20 guidelines and was recommended systematically by 17 guidelines^3,4,5,25,34,35,36,38,39,40,41,42,44,46,47,48,49^ and on a case-by-case basis without giving more details by 3 guidelines^37,43,45^ (Table 3). All guidelines recommended radiological skeletal survey up to age 2 years, except 1 guideline that did not mention any age limit,^40^ and 1 guideline^35^ that recommended it up to age 1.5 years. Twelve guidelines^3,4,5,25,34,36,39,41,42,46,48,49^ mentioned the number of views recommended in the radiological skeletal survey. The number varied from 17 views^34,36^ to 32 views,^39^ and all but 1 guideline^34^ recommended oblique views of the ribs.

**Table 3.** Imaging and Laboratory Tests Recommended in More Than 2 Included Guidelines for the Diagnostic Workup of Physical Abuse

Source	Country	Guidance target	Detection of skeletal injury			Detection of head and spine trauma					Detection of thoracic or abdominal injury			
			Radiological skeletal survey (age, y)	Follow-up skeletal survey	Bone scintigraphy	Eye fundus examination (age, y)	Head CT (age, y)	Head MRI	Spine MRI	Cranial US	Chest CT	Abdominal CT	Abdominal US	Laboratory tests^a^
New South Wales Government,^43^ 2014	Australia	Physical abuse	C	NM	C	C	C or MRI	C or CT	NM	NM	NM	C	NM	C (renal function: NM)
Government of Western Australia Department of Health,^44^ 2017	Australia	Physical abuse	S (<2)	NM	C	C (<1)	C or MRI	C or CT	NM	NM	NM	U	U	C
Canadian Paediatric Society,^46^ 2018	Canada	Inflicted skeletal injuries	S (<2)	C	C	S	U	U	NM	NM	NM	U	U	S (pancreatic enzymes: NM)
Haute Autorité de Santé,^3^ 2017	France	Shaken baby syndrome	S (<2)	C	C	S	S (<1)	S	C	No	NM	NM	C	S (urinalysis, renal function: NM)
Arbeitsgemeinschaft der Wissenschaftlichen Medizinischen Fachgesellschaften,^36^ 2019	Germany	Physical abuse	S (<2)	C	C	S (<2)	C	C	C	C	NM	NM	NM	S
Twomey et al; Children's Health Ireland,^49^ 2020	Ireland	Physical abuse^b^	S (<2)	S	NM	NE	S (<1) or C (>1)	C	C	NM	C	U	U	NE
Association of Family Physicians,^35^ 2014	Israel	Physical abuse	S (<1.5)	NM	C	S (<2)	C	NM	NM	NM	NM	C	NM	C (renal function: NM)
Japan Pediatric Society,^34^ 2014	Japan	Physical abuse	S (<2)	S	NM	S	S (<2) or MRI	S or CT	NM	NM	NM	NM	NM	NM
Nederlandse Vereniging voor Kindergeneeskunde,^42^ 2016	The Netherlands	Physical abuse	S (<2)	C	NM	C	C	C	C	NM	NM	NM	NM	NM
The Paediatric Society of New Zealand,^45^ 2016	New Zealand	Physical abuse	C (<2)	C	C	C (<2)	S (<1) or C (>1)	C	C	NM	NM	NM	NM	NM
Nasjonalt kunnskapassenter om vold og traumatisk stress,^41^ 2018	Norway	Physical abuse	S (<2)	C	No	C (<5)	S (<1) or C (1-2)	C	C	NM	C	C	NM	C
Asociación Española de Pediatría and Sociedad Española de Urgencias Pediátricas,^38^ 2010	Spain	Physical abuse	S (<2)	NM	C	S	S	C	NM	C	NM	NM	NM	C (pancreatic enzymes, urinalysis, renal function: NM)
Swedish Pediatric Society,^40^ 2019	Sweden	Physical abuse	S	S	NM	S	S	C	C	NM	C	C	NM	S (pancreatic enzymes: NM)
Swedish Pediatric Radiology Society,^39^ 2019	Sweden	Physical abuse^b^	S (<2)	S	NM	NE	S (<1) or C (>1)	C	C	NM	C	C	NM	NE
Swiss Society of Paediatrics,^37^ 2017	Switzerland	Physical abuse	C (<2)	NM	C (>2)	S	S	S	NM	NM	NM	C	C	C
Halstead et al; the Royal College of Radiologists and The Society & College of Radiographers,^5^ 2018	United Kindgom	Physical abuse^b^	S (<2)	S	No	NE	S	C	C	NM	C	S	NM	NE
Royal College of Paediatrics and Child Health,^48^ 2019	United Kingdom	Physical abuse	S (<2)	S	C	S	S (<1) or 1 C (1-2)	C	C	No	NM	C	NM	C (urinalysis: NM)
Christian and the Committee on Child Abuse and Neglect, American Academy of Pediatrics,^4^ 2015	United States	Physical abuse	S (<2)	C	C	S	C	C	C	C	NM	C	NM	S (urinalysis: C, renal function: NM)
Wootton-Gorges et al; Expert Panel on Pediatric Imaging, American College of Radiology,^25^ 2017	United States	Physical abuse^b^	S (<2)	C	C	NE	S	C	S	NM	C	S	No	NE
Narang et al; the Council on Child Abuse and Neglect, American Academy of Pediatrics,^47^ 2020	United States	Abusive head trauma	S (<2)	NM	NM	S	S or MRI	S or CT	C	No	NM	NM	NM	NM

Abbreviations: C, case by case basis; CT, computed tomography; NM, not mentioned; MRI, magnetic resonance imaging; NE, not expected; S, systematic; U, unclear; US, ultrasonography.

^a^ Includes pancreatic enzymes, liver enzymes, urinalysis, and renal function.

^b^ Detected using imaging tests.

Bone scintigraphy was mentioned in 14 of 20 guidelines (70%). It was recommended on a case-by-case basis by 12 guidelines,^3,4,25,35,36,37,38,43,44,45,46,48^ for children 2 years and older by 1 guideline,^37^ as a complementary test in case of negative radiological skeletal survey results and if the suspicion of abuse remained high by 10 guidelines,^3,4,25,35,36,38,44,45,46,48^ without giving more details by 1 guideline,^43^ and not recommended by 2 guidelines.^5,41^

Of 20 guidelines, 14 (70%) mentioned follow-up radiological skeletal survey (recommended to be performed between 7 days and 3 weeks after the first survey; 14 days for most of the guidelines), including systematically by 6 guidelines^5,34,39,40,48,49^ and only in case of doubt in the initial investigation by 8 guidelines.^3,4,25,36,41,42,45,46^ The number of recommended views was mentioned in 6 guidelines^3,5,25,34,36,46,49^ and varied from 9 views^5^ to 17 views.^34^

#### Detection of Head and Spine Trauma

All 20 guidelines gave a recommendation regarding diagnostic tests to be performed for head and spine trauma. All 16 expected guidelines recommended an eye fundus examination, systematically^3,4,34,35,36,37,38,40,46,48^ or on a case-by-case-basis according to the clinical context.^41,42,43,44,45^ Only 5 guidelines recommended the upper age limit to perform an eye fundus examination, which ranged from 1 year^35,36,44,45^ to 5 years^41^ (Table 3).

All guidelines recommended a head CT, including 13 guidelines^3,5,25,34,37,38,39,40,45,47,48,49^ that recommended it systematically (age <1 year for 6 guidelines^3,39,41,45,48,49^) and 6 guidelines^4,35,36,42,43,44^ that recommended it on a case-by-case basis; 1 guideline^46^ was unclear by mentioning *neuro-imaging* without giving more details. The use of contrast product injection was mentioned in 7 guidelines^3,25,35,39,41,45,49^: all recommended not using it. Of 20 expected guidelines, 19 guidelines (95%) recommended a head MRI, including 12 guidelines^4,5,25,34,36,39,40,41,42,45,48,49^ that recommended head MRI if head CT found anomalous results, 2 guidelines^3,37^ that recommended systematically performing both head CT and head MRI whatever the initial results of head CT, and 3 guidelines^43,44,47^ that recommended performing any of these 2 tests. Ten guidelines^3,4,5,25,34,36,39,41,45,48^ detailed the procedure for performing head MRI and were consistent on the performance of diffusion-weighted imaging but differed in the details of the sequences to be performed.

Spinal MRI was mentioned in 13 guidelines (65%). Performance of spinal MRI was recommended as systematic by 1 guideline,^25^ in case of diagnostic concern by 2 guidelines,^3,47^ and only if a head MRI was performed by 10 guidelines.^4,5,36,39,40,41,42,45,48,49^ One guideline^41^ recommended only a cervical spine MRI, 2 guidelines^4,47^ were unclear about recommending cervical or complete spine MRI, and all other guidelines recommended a complete spine MRI.^3,5,25,36,39,40,42,45,48,49^

Cranial ultrasonography was mentioned in 6 guidelines (30%). It was recommended for the evaluation of macrocephaly,^4^ if a skull injury is suspected,^36^ or without giving more detail,^38^ and was not recommended by 3 guidelines.^3,47,48^

#### Detection of Thoracoabdominal Injuries

Of 20 guidelines, 16 guidelines (80%) gave a recommendation regarding which diagnostic tests should be performed for thoracoabdominal injuries. Chest CT was mentioned in 6 guidelines^5,25,39,40,41,49^ (22%) (Table 3); it was recommended if needed for evaluating rib injuries in all of the guidelines. Abdominal CT was mentioned in 10 guidelines (50%): as systematic by 2 guidelines^5,25^ and on a case-by-case basis by 8 guidelines^4,35,37,39,40,41,43,48^ conditional to the laboratory tests results, in case of symptomatic^4,40^ or suspected abdominal injuries^39,41,48^ or without giving more details.^35,37,43^ Four guidelines^4,5,25,41^ mentioned the use of contrast product injection for abdominal CT and recommended it, and 3 guidelines^44,46,49^ recommended performing abdominal imaging without giving more details on the imaging procedures. Abdominal ultrasonography was mentioned in 3 guidelines (15%), with 2 guidelines^3,37^ recommending it according to the clinical context without giving more details, and 1 guideline^25^ not recommending it.

In total, 12 of 16 expected guidelines^3,4,35,36,37,38,40,41,43,44,46,48^ (75%) listed the laboratory tests to be performed for thoracoabdominal injuries (ie, liver enzymes, pancreatic enzymes, urinalysis, and renal function). All 4 tests were recommended systematically by 1 guideline,^36^ and according to the clinical context without giving more details by 3 guidelines^35,41,44^; 2 guidelines^40,46^ recommended systematic performance of liver enzymes, urinalysis, and renal function testing only, 2 guidelines^3,4^ recommended systematic performance of liver and pancreatic enzymes, and 1 guideline^38^ recommended testing liver enzymes “in case of bruises or muscle injuries” (without specifying their location). Six guidelines recommended renal function testing systematically,^36,40,46^ or according to the clinical context without giving more details.^37,41,44^ Four guidelines recommended troponin and/or creatine kinase testing to detect cardiac injury systematically^4,36,40^ or according to the clinical context^41^ without giving more details, but the other guidelines did not mention these tests.

#### Differential Diagnosis

Of 16 expected guidelines, 9 guidelines^3,4,34,35,36,37,38,40,41,42,43,44,45,46,47^ (56%) gave a recommendation regarding laboratory tests to be performed to explore bone metabolism (Table 4). Seven guidelines recommended serum calcium, phosphorus, and alkaline phosphatase tests systematically^4,36,40,46^ or according to the clinical context without giving more details^41,43,48^; 1 guideline^37^ recommended only serum calcium tests according to the clinical context without giving more details. Seven guidelines recommended parathyroid hormone and 25-hydroxy-vitamin D laboratory tests systematically^36,40^ or according to the clinical context without giving more details.^4,41,43,46,48^ Five guidelines recommended serum copper and ceruloplasmin systematically^36,40^ or according to the clinical context without giving more details.^4,41,46^ One guideline^48^ mentioned serum copper test without recommending it clearly. Three guidelines^4,41,48^ recommended fibroblast culture and/or DNA analysis in case of suspicion of osteogenesis imperfecta.

**Table 4.** Laboratory Blood Tests Recommended for Differential Diagnoses, Such as Bone Fragility and Bleeding Disorders in Guidelines

Source	Country	Bone fragility					Bleeding disorders			Subdural hematoma	
		Calcium, phosphorus, alkaline phosphatase	25-hydroxyvitamin D, PTH	Serum copper, ceruloplasmin, and vitamin C^a^	Fibroblast culture and/or DNA analysis for osteogenesis imperfecta	CBC with platelets, PT/INR/aPTT/fibrinogen		Factor VIII/factor IX/VWF activity	Urine organic acids^b^	
New South Wales Government,^43^ 2014	Australia	C (with magnesium)	C	NM	NM	C (PT and fibrinogen: NM)		U	C	
Government of Western Australia Department of Health,^44^ 2017	Australia	NM	NM	NM	NM	C (coagulation: U)		NM	NM	
Canadian Paediatric Society,^46^ 2018	Canada	S	C	C (vitamin C: NM)	NM	S (coagulation: NM)		NM	NM	
Haute Autorité de Santé,^3^ 2017	France	NM	NM	NM	NM	S (INR: NM; with lactates)		S (with factor XI)	C	
Arbeitsgemeinschaft der Wissenschaftlichen Medizinischen Fachgesellschaften,^36^ 2019	Germany	S	S	S	NM	S (INR: NM)		C (with factor XIII and blood group)	C	
Association of Family Physicians,^35^ 2014	Israel	NM	NM	NM	NM	C (coagulation: U)		NM	NM	
Japan Pediatric Society,^34^ 2014	Japan	NM	NM	NM	NM	NM		NM	NM	
Nederlandse Vereniging voor Kindergeneeskunde,^42^ 2016	The Netherland	NM	NM	C (serum copper, and ceruloplasmine: NM)	NM	C (INR: NM)		C (with factor XIII)	NM	
The Paediatric Society of New Zealand,^45^ 2016	New Zealand	NM	NM	NM	NM	C (PT and fibrinogen: NM)		C (factor VIII, factor IX: NM, with blood group)	C	
Nasjonalt kunnskapassenter om vold og traumatisk stress,^41^ 2018	Norway	C (with Na)	C	C (vitamin C: NM)	C	C (PT: NM)		C (with factor VII and vitamin K)	C	
Asociación Española de Pediatría and Sociedad Española de Urgencias Pediátricas,^38^ 2010	Spain	NM	NM	NM	NM	S (coagulation: U)		NM	NM	
Swedish Pediatric Society,^40^ 2019	Sweden	S (with Na and K)	S	S (vitamin C: NM)	NM	S (with lactates)		S	NM	
Swiss Society of Paediatrics,^37^ 2017	Switzerland	C (phosphorus, alkaline phosphatase: NM; with Na, K, and Cl)	NM	NM	NM	C (aPTT: NM)		C (VWF activity: NM, with factor XIII)	C	
Royal College of Paediatrics and Child Health,^48^ 2019	United Kingdom	C	C	U (ceruloplasmin: NM, with vitamin A)	C	C		C (factor IX and factor XIII)	C	
Christian and the Committee on Child Abuse and Neglect, American Academy of Pediatrics,^4^ 2015	United States	S	C	C	C	S		S (with D-dimer)	C	
Narang et al; the Council on Child Abuse and Neglect, American Academy of Pediatrics,^47^ 2020	United States	NM	NM	NM	NM	NM		NM	NM	

Abbreviations: aPTT, activated partial thromboplastin time; C, case by case basis; CBC, complete blood cell count; Cl, chloride; D-dimer, dimerized plasmin fragment D; INR, international normalized ratio; K, Potassium; Na, sodium; NM, not mentioned; PT, prothrombin time; PTH, parathyroid hormone; S, systematic; VWF, von Willebrand factor.

^a^ Performed for the detection of Menkes disease and scurvy.

^b^ Performed for the detection of glutaric aciduria type 1.

Of 16 expected guidelines, 14 guidelines^3,4,35,36,37,38,40,41,42,43,44,45,46,48^ (88%) gave a recommendation regarding laboratory tests to be performed to explore bleeding disorders. Fourteen guidelines recommended a complete blood count, including platelets systematically^3,4,36,38,40,46^ or according to the clinical context^35,37,41,42,43,44,45,48^ without giving more details, except for 2 guidelines^44,48^ that recommended these tests in case of bruises. Three guidelines recommended a regular coagulation test (ie, activated partial thromboplastin time, prothrombin time, international normalized ratio, and fibrinogen) systematically^4,40^ or in case of bruises^48^; 7 guidelines^3,36,37,41,42,43,45^ recommended a regular coagulation test without mentioning all these tests. Three guidelines^35,38,44^ recommended a coagulation screening test without giving details on the tests to be performed. Seven guidelines recommended advanced coagulation tests (ie, factor VIII and IX levels and von Willebrand activity with or without factor XI and XIII levels) systematically^3,4,40^ or according to the clinical context^36,40,41,42^ without giving more details. One guideline^45^ recommended testing von Willebrand activity in case of blood disorder suspicion, and 1 guideline^43^ recommended a full coagulation testing without giving details on the tests to be performed. Eight guidelines^3,4,36,37,41,43,45,48^ recommended to test urine organic acids in case of suspicion of glutaric aciduria type 1.

## Discussion

In this systematic review of 20 clinical guidelines for the early detection and diagnostic workup of CPA in infants, we identified a few unclear statements but a frequent lack of completeness of guidelines and numerous between-guideline discrepancies. Guidelines agreed with recommending radiological skeletal survey, head CT, head MRI, and eye fundus examination but disagreed on whether these should be systematically performed or not. Other main discrepancies dealt with defining sentinel injuries and performing bone scintigraphy, follow-up skeletal survey, spinal MRI, cranial ultrasonography, chest CT, and abdominal ultrasonography and CT systematically, on case-by-case basis, or not. For ruling out differential diagnoses, guidelines agreed on blood tests to explore primary hemostasis and coagulation, but with some discrepancies in the tests to be performed, and only half of the included guidelines mentioned the need to investigate bone metabolism.

The guidelines were based on a limited number of well-designed primary studies, particularly for the definition and diagnosis of sentinel injuries,^14,50^ the use of bone scintigraphy,^20^ and laboratory tests^29,51^ to perform in case of suspected CPA. The lack of primary studies may have led guidelines developers to opt for expert consensus rather than evidence-based guidelines, and this may explain the substantial heterogeneity among guidelines. Also, the methods used by the developers of these guidelines often did not follow international recommendations.^32,33^ Only 4 guidelines provided details on the quality of the underlying evidence and rated the strength of their statements, even though these steps are known to improve guidelines’ implementation.^52^ The high rate of missing statements (41%) could be explained by a failure to describe the precise scope of the guidelines, such that their authors did not indicate which tests should not be performed. For example, the limitations of cranial ultrasonography to detect inflicted brain injuries are well known, although this test was still recommended in 2 guidelines. Thus, the nonindication for cranial ultrasonography probably needs to be reaffirmed in guidelines to avoid misleading nonspecialized clinicians. More generally, the scope of guidelines should be to state both recommended and not recommended imaging and laboratory tests for the diagnostic workup of CPA.

### Interpretation and Implications

The lack of completeness of guidelines and between-guideline inconsistencies may mislead physicians’ decisions. Efforts at the national level to standardize practices by producing guidance to help physicians optimize the detection of inflicted injuries and consider differential diagnoses may be jeopardized by the heterogeneity observed among guidelines at the international level, given their high online accessibility. Between-country variability of clinical guidance may be explained by regional variations in the epidemiological characteristics of diseases or accessibility of diagnostic tests,^53^ but the detection and the diagnostic workup of CPA in high-income countries should be standardized. Other factors, such as clinical recommendations published in journals with high impact factor,^9^ could also influence physicians’ decisions, but we believe this between-guideline heterogeneity explains in part the variability and the suboptimality of observed practices for the detection and diagnostic workup of CPA.^16,17,54^

Our systematic review could aid in drawing the research agenda to optimize the detection and diagnostic workup of CPA. First, priority clinical questions for which guidelines lacked consistency were bone scintigraphy, follow-up skeletal survey, spine MRI, cranial ultrasonography, chest CT, abdominal CT and ultrasonography, and laboratory tests for abdominal injuries or for differential diagnoses. The methods needed to reach international consensus may vary depending on the clinical question. For example, in the past decade, well-designed original studies and systematic reviews showed the important role of head and spine MRI to detect additional and extracranial injuries.^55,56,57,58,59^ Thus, an update of the oldest included guidelines would probably lead to more between-guideline consistency. Other systematic reviews have shown the complete lack of well-designed original studies, such as for bone scintigraphy,^20^ pointing to the need to conduct such studies. There is also a clear need to define sentinel injuries, to agree not just on their location, size, patterns, and number but also on the term *sentinel injury* because it is not shared by all experts in the field of CPA. The TEN-4 rule could help in the definition of sentinel injuries by providing a simple tool to help clinicians classify bruises as sentinel injuries.^15^ Finally, our results suggest that developers of guidelines for CPA detection and diagnosis should follow international recommendations for their development process to notably rate the strength of recommendations based on the available evidence. Given the constant production of knowledge in the detection and diagnostic workup of CPA, an international consensus should be actualized on a regular basis to incorporate all the available evidence, as has been done for abusive head trauma with a consensus statement supported by 15 major national and international professional medical societies.^60^

### Limitations

This study has some limitations. First, we could not find guidelines in more than one-third of the high-income countries included in the search, even though we performed a systematic search of several databases and relevant websites, with no language restriction. We could only identify 4 additional guidelines by asking European experts in pediatrics what guidelines were in force in their country. We may have missed existing guidelines, in particular in countries not covered by the ECPCP. However, an exhaustive search would probably have increased the between-guideline variability. Second, we subjectively decided the specific guidance expected for each guideline according to the title and scope, by a consensus of coauthors, to compare them. Third, by removing the tests suggested in 2 guidelines or fewer, we risked not analyzing promising new tests, such as whole-body MRI for the detection of skeletal and muscular injuries.^61^

## Conclusions

This systematic review identified flaws in guidelines’ completeness and between-guideline discrepancies that could contribute to the observed variations in clinical practices. Primary care health practitioners and hospital-based physicians are the first-line and key actors for the early detection and diagnosis of CPA in infants, and their decisions should be based on complete, clear, and consistent guidelines. Our findings may help identify priorities for well-designed original diagnostic accuracy studies, systematic reviews or an international consensus process to produce clear and standardized guidelines to optimize practices and infants’ outcomes.

## Notes

Supplement 1. **eTable 1.** Countries for Which Guidelines for the Diagnostic Workup for Child Physical Abuse Were Specifically Searched on Website of the United Nations **eTable 2.** Search Strategy **eFigure.** Flowchart of Guidelines in the Systematic Review **eTable 3.** Recommended Imaging and Laboratory Tests for Suspicion of Physical Abuse **eTable 4.** Guidance Expected Given the Scope of the 20 Guidelines Included in the Systematic Review **eTable 5.** Expected and Not Expected Statements Given the Scope of Guidelines for the Diagnosis of Child Abuse
eReferences

Supplement 2.
Group Information
