# Supplementary material for: Variations in Guidelines for Diagnosis of Child Physical Abuse in High-Income Countries: A Systematic Review
Source: JAMA Netw Open. 2021 Nov 17;4(11):e2129068. doi: 10.1001/jamanetworkopen.2021.29068 (PMC8600386; doi:10.1001/jamanetworkopen.2021.29068)
Supplement: Supplement 1. — eTable 1. Countries for Which Guidelines for the Diagnostic Workup for Child Physical Abuse Were Specifically Searched on Website of the United Nations eTable 2. Search Strategy eFigure. Flowchart of Guidelines in the Systematic Review eTable 3. Recommended Imaging and Laboratory Tests for Suspicion of Physical Abuse eTable 4. Guidance Expected Given the Scope of the 20 Guidelines Included in the Systematic Review eTable 5. Expected and Not Expected Statements Given the Scope of Guidelines for the Diagnosis of Child Abuse eReferences [file jamanetwopen-e2129068-s001.pdf]

Supplemental Online Content

Blangis F, Allali S, Cohen JF, et al; European Confederation of Primary Care Paediatricians (ECPCP) research group. Variations in guidelines for diagnosis of child physical abuse in high-income countries: a systematic review. *JAMA Netw Open*. 2021;4(11):e2129068. doi:10.1001/jamanetworkopen.2021.29068

eTable 1. Countries for Which Guidelines for the Diagnostic Workup for Child Physical Abuse Were Specifically Searched on Website of the United Nations

eTable 2. Search Strategy

eFigure. Flowchart of Guidelines in the Systematic Review

eTable 3. Recommended Imaging and Laboratory Tests for Suspicion of Physical Abuse

eTable 4. Guidance Expected Given the Scope of the 20 Guidelines Included in the Systematic Review

eTable 5. Expected and Not Expected Statements Given the Scope of Guidelines for the Diagnosis of Child Abuse

eReferences

This supplemental material has been provided by the authors to give readers additional information about their work.

© 2021 Blangis F et al. *JAMA Network Open.*

eTable 1. Countries for Which Guidelines for the Diagnostic Workup for Child Physical Abuse Were Specifically Searched on Website of the United Nations

We restricted the research to countries with advanced economies according to the World Economic Situation and Prospects classification[1] and we added Israel.

**Covered by the ECPCP**[a]		**Not covered by the ECPCP**
Austria		Australia
Finland		Belgium
France		Canada
Germany		Denmark
Israel		Greece
Italy		Iceland
Luxembourg		Ireland
Portugal		Japan
Spain		Netherlands
Switzerland		New Zealand
		Norway
		Sweden
		United Kingdom
		United States

[a] European Confederation of Primary Care Paediatricians

© 2021 Blangis F et al. *JAMA Network Open.*

eTable 2. Search Strategy
(last update on June 2020)

a- Medline

1	((((("Child Abuse"[Mesh] OR "child abuse[tiab]" OR "abused child[tiab]" OR "Battered Child Syndrome"[Mesh] OR "battered child syndrome[tiab]"OR "battered children[tiab]" OR "Wounds and Injuries"[Mesh] OR "non-accidental injury[tiab]") AND (Diagnosis[Mesh] OR diagnostic[tiab] OR diagnosis[tiab] OR investigation[tiab]) AND ("infant"[MeSH Terms] OR "child"[MeSH Terms] OR "adolescent"[MeSH Terms]) AND (Guideline[ptyp]) AND ("2000/01/01"[PDAT] : "2020/03/31"[PDAT])))))	114

b- Web of Science

1	TS: ((("child abuse" OR "abused child" OR "battered child syndrome" OR "battered children" OR "non-accidental injury") AND (diagnostic OR diagnosis OR investigation) AND (Guidelines OR recommendations))) OR TI: ((("child abuse" OR "abused child" OR "battered child syndrome" OR "battered children" OR "non-accidental injury") AND (diagnostic OR diagnosis OR investigation) AND (Guidelines OR recommendations))) Indexes=SCI-EXPANDED, SSCI, A&HCI, CPCI-S, CPCI-SSH, BKCI-S, BKCI-SSH, ESCI Timespan: 2000-2020	153

c- Google Scholar

The search strategy on Google Scholar was “physical abuse” “diagnosis” “detection” “guideline” and “child” -published since 2000-, limiting the analysis to the first 500 results.

© 2021 Blangis F et al. *JAMA Network Open.*

eFigure. Flowchart of Guidelines in the Systematic Review

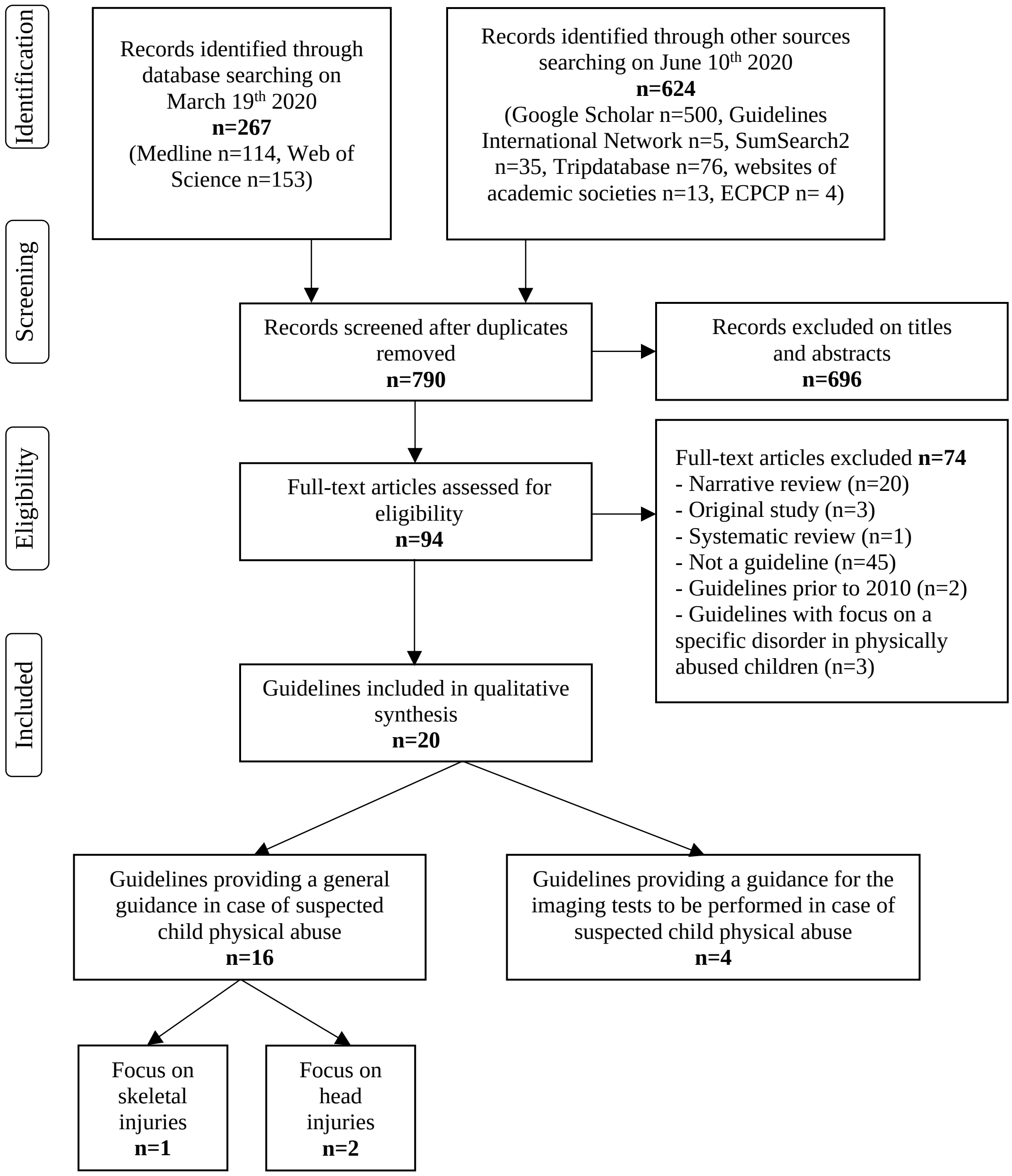

© 2021 Blangis F et al. *JAMA Network Open.*

eTable 3. Recommended Imaging and Laboratory Tests for Suspicion of Physical Abuse

**Source**	**New South Wales Government[2]**	**Government of Western Australia Department of Health[3]**	**Canadian Paediatric Society[4]**	**Haute Autorité de Santé[5]**	**Arbeitsgemeinschaft der Wissenschaftlichen Medizinischen Fachgesellschaften[6]**
Country	**Australia**	**Australia**	**Canada**	**France**	**Germany**
Year	**2014**	**2017**	**2018**	**2017**	**2019**
Objective for guidance	Physical abuse	Physical abuse	Inflicted skeletal injuries	Shaken baby syndrome	Physical abuse
	Definition of sentinel injuries				
**Type**	Yes	Yes	Yes	Yes	Yes
**Location**	NM	Yes	Yes	Yes	NM
**Number**	NM	Yes	NM	NM	NM
**Size**	NM	NM	NM	NM	NM
**Pattern**	NM	Yes	NM	NM	NM
**According to child age and/or if cruising child**	NM	Yes	Yes	Yes	NM
	Detection of skeletal or soft tissue injury				
**Radiological skeletal survey**	C	S <2 years	S <2 years	S <2 years	S <2 years
Number of views	NM	NM	24	19	17-19
Views	NM	NM	Frontal and lateral views of the skull, lateral views of the cervical spine and thoracolumbosacral spine, and single frontal views of the long bones, hands, feet, chest, and abdomen, oblique views of the ribs	S: frontal and lateral views of the skull (if no CT scan), frontal and lateral views of the spine, frontal view of the pelvis, frontal and lateral views of the chest, frontal views of each limb segment<br>C: lateral views of the knees and ankles	Frontal and lateral views of the skull, frontal view of the chest, frontal views of the upper arms, forearms and hands, frontal views of the thighs, lower legs and feet<br>If no rib fractures are detected:<br>Lateral views of the chest<br>If detection of one or more spinal fractures laterally, frontal view of the abdomen with pelvis and hips
**Follow-up skeletal survey**	NM	NM	C (14 days)	C (15 days)	C (11 to 14 days)
Number of views	NM	NM	10 minimum	13	16
Views	NM	NM	Omitting images of the skull, pelvis and lateral spine	S: frontal view of the pelvis, frontal and lateral views of the chest, frontal views of each limb segment<br>C: lateral views of the knees and ankles	Frontal and lateral views of the skull, frontal view of the chest, frontal views of the upper arms, forearms and hands, frontal views of the thighs, lower legs and feet
**Bone scintigraphy**	C	C	C	C	C (ribs)
Sedation	NM	NM	NM	NM	NM
**Ultrasonography (bones)**	NM	NM	NM	NM	NM
**Whole-body MRI**	NM	NM	NM	NM	NM
With contrast	NM	NM	NM	NM	NM
Sedation	NM	NM	NM	NM	NM
	Detection of head or spine injury				
**Eye fundus examination**	C	C <1 year	S	S	S < 2 years
Number, type, extent, patterns of retinal haemorrhages	NM	NM	Yes	NM	Yes

© 2021 Blangis F et al. *JAMA Network Open.*

eTable 3. Recommended Imaging and Laboratory Tests for Suspicion of Physical Abuse (continued)

Source	New South Wales Government[2]	Government of Western Australia Department of Health[3]	Canadian Paediatric Society[4]	Haute Autorité de Santé[5]	Arbeitsgemeinschaft der Wissenschaftlichen Medizinischen Fachgesellschaften[6]
**Head CT**	C	C	U	S <1 year	C (vital risk)
With contrast	NM	NM	NM	No	NM
**Head MRI**	C	C	U	S	C (no vital risk)
Abnormal CT scan	NM	NM	NM	Yes	Yes
Suspicion of abuse without neurological symptoms	NM	NM	NM	NM	NM
Sequences	NM	NM	NM	At least axial and sagittal T1 weighted images, axial T2 weighted images, diffusion weighted images and T2*	Diffusion weighted images
With contrast	NM	NM	NM	NM	NM
Sedation	NM	NM	NM	NM	NM
**Spinal MRI**	NM	NM	NM	C	C
Complete spine	NM	NM	NM	C	Yes
**Cranial ultrasonography**	NM	NM	NM	No	C
**EEG**	NM	NM	NM	S	NM
	Detection of thoracic or abdominal injury				
**Chest CT**	NM	NM	NM	NM	NM
With contrast	NM	NM	NM	NM	NM
**Abdominal ultrasonography**	NM	U	U	C	NM
**Abdominal CT**	C	U	U	NM	NM
With contrast	NM	NM	NM	NM	NM
**Pelvis CT**	NM	NM	NM	NM	NM
With contrast	NM	NM	NM	NM	NM
**Laboratory tests**					
Liver enzymes	C	C	S	S	S
Pancreatic enzymes	C	C	NM	S	S
Urinalysis	C (+toxicology)	C	S	NM	S
Renal function	NM	NM	S	NM	S
Troponin, creatine kinase	NM	C	NM	NM	S (creatine kinase)
	Differential diagnosis				
Calcium, phosphorus, alkaline phosphatase	C (+ magnesium)	NM	S	NM	S
25-hydroxyvitamin D, PTH	C	NM	C	NM	S
Skin biopsy for fibroblast culture and/or venous blood for DNA analysis for osteogenesis imperfecta	NM	NM	NM	NM	NM
CBC with platelets, coagulation regular (PT/INR/aPTT/fibrinogen)	C (PT, fibrinogen: NM)	C (coagulation: U)	S (coagulation: NM)	S (INR: NM; +lactates)	S (INR: NM)
Coagulation advanced (factor VIII level/factor IX level/VWF activity)	U	NM	NM	S (+factor XI)	C (+ factor XIII and blood group)
Serum copper, ceruloplasmin and vitamin C levels	NM	NM	C (vitamin C:NM)	NM	S
Urine organic acids[a]	C	NM	NM	C	C
New-born screen review	NM	NM	NM	NM	NM

CT, computed tomography; MRI, magnetic resonance imaging; EEG = electroencephalography
PTH, parathyroid hormone; CBC, complete blood cell count; PT, prothrombin time; INR, international normalized ratio; aPTT, activated partial thromboplastin time; VWF, von Willebrand factor
S, systematic; NM, not mentioned; C, case-by-case basis; U, unclear
[a] Performed for the detection of glutaric aciduria type 1

© 2021 Blangis F et al. *JAMA Network Open.*

eTable 3. Recommended Imaging and Laboratory Tests for Suspicion of Physical Abuse (continued)

Source	Children's Health Ireland[7]	Association of Family Physicians[8]	Japan Pediatric Society[9]	Nederlandse Vereniging voor Kindergeneeskunde[10]	Starship / The Paediatric Society of New Zealand[11]
Country	**Ireland**	**Israel**	**Japan**	**Netherland**	**New Zealand**
Year	**2020**	**2014**	**2014**	**2016**	**2016**
Objective for guidance	Physical abuse (imaging tests)	Physical abuse	Physical abuse	Physical abuse	Physical abuse
	Definition of sentinel injuries				
**Type**	NE	Yes	Yes	Yes	Yes
**Location**	NE	Yes	NM	Yes	Yes
**Number**	NE	NM	NM	Yes	NM
**Size**	NE	Yes	NM	Yes	NM
**Pattern**	NE	Yes	NM	Yes	Yes
**According to child age and/or if cruising child**	NE	NM	NM	Yes	Yes
	Detection of skeletal or soft tissues injury				
**Radiological skeletal survey**	S <2 years	S <36 months	S <2 years	S <2 years	C <2 years
Number of views	25	NM	17	21 to 24	NM
Views	Frontal and bilateral oblique views of the chest (3); abdomen and pelvis (1); frontal views of the upper and lower extremities, either each limb on a single view or more commonly two exposures for each limb (8); hands and feet (4); lateral spine (1); coned lateral views of the elbows, wrists, knees and ankles (8)	Spine, limbs and skull	Frontal view of the skull, frontal view of the thorax, lateral view of the cervical spine, lateral view of the lumbar spine, frontal view of the pelvis, frontal views of the upper and lower limbs, frontal view of the arms, frontal views of the forearm, frontal views of the hands, frontal views of the feet	Skull: AP and lateral Front suboccipital image, if clinically indicated Thorax: AP, including clavicles. Three quarters, front left and right front Abdomen: AP, including pelvis and hips Vertebral column: Lateral, entire spine (in multiple shots if needed). AP, if not imaged on thorax and abdomen. AP of the cervical spine (if indicated) Extremities: AP upper arms, AP forearms, AP thighs, AP lower legs, PA hands, AP feet.	NM
**Follow-up skeletal survey**	S (11 to 14 days)	NM	S (7 to 14 days)	C	C (14 days)
Number of views	11	NM	17	NM	NM
Views	This can be limited to frontal and bilateral oblique views of the chest, frontal views of the limbs and follow up of positive, suspected or equivocal findings on the primary skeletal survey. A repeat of the full skeletal survey may be undertaken if there is significant concern for occult injuries.	NM	Idem RSC → Skull front side, thorax (not chest) front side, cervical side, lumbar side, hip joint front, femur front (left and right) Front of lower leg (right and left) Front of humerus, front of forearm, front of phalange, front of toes	NM	NM
**Bone scintigraphy**	NM	C	NM	NM	C
Sedation	NM	NM	NM	NM	NM
**Ultrasonography (bones)**	NM	NM	NM	NM	NM
**Whole-body MRI**	NM	NM	NM	NM	NM
With contrast	NM	NM	NM	NM	NM
Sedation	NM	NM	NM	NM	NM
	Detection of head or spine injury				
**Eye fundus examination**	NE	S < 2 years	S	C	C <2 years
Number, type, extent, patterns of retinal haemorrhages	NE	NM	Yes	NM	NM

© 2021 Blangis F et al. *JAMA Network Open.*

eTable 3. Recommended Imaging and Laboratory Tests for Suspicion of Physical Abuse (continued)

Source	Children's Health Ireland[7]	Association of Family Physicians[8]	Japan Pediatric Society[9]	Nederlandse Vereniging voor Kindergeneeskunde[10]	Starship / The Paediatric Society of New Zealand[11]
**Head CT**	S <1 year<br>C >1 year	C	S < 2 years (or MRI)	C	S <1 year<br>C >1 year
With contrast	No	No	NM	NM	No
**Head MRI**	C	NM	S (or CT)	C	C
Abnormal CT scan	Yes	NM	Yes	Yes	Yes
Suspicion of abuse without neurological symptoms	NM	NM	NM	Yes	Yes
Sequences	NM	NM	Including diffusion weighted images	NM	Gradient echo sequences, diffusion-weighted images and susceptibility-weighted images
With contrast	NM	NM	NM	NM	NM
Sedation	NM	NM	U	NM	NM
**Spinal MRI**	C	NM	NM	C	C
Complete spine	Yes	NM	NM	Yes	Yes
**Cranial ultrasonography**	NM	NM	NM	NM	NM
**EEG**	NE	NM	NM	NM	NM
	Detection of thoracic or abdominal injury				
**Chest CT**	C	NM	NM	NM	NM
With contrast	NM	NM	NM	NM	NM
**Abdominal ultrasonography**	U	NM	NM	NM	NM
**Abdominal CT**	U	C	NM	NM	NM
With contrast	NM	NM	NM	NM	NM
**Pelvis CT**	NM	NM	NM	NM	NM
With contrast	NM	NM	NM	NM	NM
**Laboratory tests**					
Liver enzymes	NE	C	NM	NM	NM
Pancreatic enzymes	NE	C	NM	NM	NM
Renal function	NE	NM	NM	NM	NM
Urinalysis	NE	C (+toxicology)	NM	NM	NM
Troponin, creatine kinase	NE	NM	NM	NM	NM
	Differential diagnosis				
Calcium, phosphorus, alkaline phosphatase	NE	NM	NM	NM	NM
25-hydroxyvitamin D, PTH level	NE	NM	NM	NM	NM
Skin biopsy for fibroblast culture and/or venous blood for DNA analysis for osteogenesis imperfecta	NE	NM	NM	NM	NM
CBC with platelets, coagulation regular (PT/INR/aPTT/fibrinogen)	NE	C (coagulation: U)	NM	C (INR: NM)	C (PT, fibrinogen: NM)
Coagulation advanced (factor VIII level/factor IX level/VWF activity)	NE	NM	NM	C (+ factor XIII)	C (VWF activity + blood group)
Serum copper, ceruloplasmin and vitamin C levels	NE	NM	NM	C (vitamin C)	NM
Urine organic acids[a]	NE	NM	NM	NM	C
New-born screen review	NE	NM	NM	NM	NM

CT, computed tomography; MRI, magnetic resonance imaging; EEG = electroencephalography
PTH, parathyroid hormone; CBC, complete blood cell count; PT, prothrombin time; INR, international normalized ratio; aPTT, activated partial thromboplastin time; VWF, von Willebrand factor
S, systematic; NM, not mentioned; C, case-by-case-basis; NE, not expected; U, unclear
[a] Performed for the detection of glutaric aciduria type 1

© 2021 Blangis F et al. *JAMA Network Open.*

**eTable 3.** Recommended Imaging and Laboratory Tests for Suspicion of Physical Abuse (continued)					
**Source**	**Nasjonalt kunnskapassenter om vold og traumatisk stress[12]**	**Asociación Española de Pediatría and Sociedad Española de Urgencias Pediátricas[13]**	**Swedish Pediatric Society[14]**	**Swedish Paediatric Radiology Society[15]**	**Swiss Society of Paediatrics[21]**
Country	**Norway**	**Spain**	**Sweden**	**Sweden**	**Switzerland**
Year	**2018**	**2010**	**2019**	**2019**	**2017**
Objective for guidance	Physical abuse	Physical abuse	Physical abuse	Physical abuse (imaging tests)	Physical abuse
	Definition of sentinel injuries				
**Type**	Yes	Yes	Yes	NE	Yes
**Location**	Yes	NM	NM	NE	NM
**Number**	No	Yes	NM	NE	NM
**Size**	No	NM	NM	NE	NM
**Pattern**	No	NM	NM	NE	NM
**According to child age and/or if cruising child**	No	NM	NM	NE	NM
	Detection of skeletal or soft tissues injury				
**Radiological skeletal survey**	S <2 years	S <2 years	S	S <2 years	C <2 years
Number of views	22	NM	NM	28-32	NM
Views	Skull - front, sides (both sides towards detector), possibly Townes projection; should be performed in anyone under two years of age (even if cerebral CT has been performed)<br>Thorax skeleton, front, side, oblique (skeletal exposure)<br>Whole column (C-Th-LS), side<br>Abdomen including pelvis, front<br>Upper and forearms (AP), hands (PA)<br>Thighs, calves and feet (AP)	NM	NM	Frontal and lateral views of the skull, frontal view and lateral views of the chest, frontal view of the abdomen and pelvis, lateral view of the spine, frontal view of the upper arms, forearms, elbows and hand, frontal view of the upper and lower limbs, knees, ankles, feet	NM
**Follow-up skeletal survey**	C (11-14 days)	NM	S (14 days)	S (11-14 days)	NM
Number of views	NM	NM	NM	NM	NM
Views	NM	NM	Whole-body skeleton X-ray	NM	NM
**Bone scintigraphy**	No	C	NM	NM	C > 2 years
Sedation	NM	NM	NM	NM	NM
**Ultrasonography (bones)**	NM	NM	NM	NM	NM
**Whole-body MRI**	No	NM	NM	NM	NM
With contrast	NM	NM	NM	NM	NM
Sedation	NM	NM	NM	NM	NM
	Detection of head or spine injury				
**Eye fundus examination**	C <5 years old	S	S	NE	S
Number, type, extent, patterns of retinal haemorrhages	Yes	NM	Yes	NE	NM
**Head CT**	S <1 year, C 1 to 2 years old	S	S	S <1 year<br>C >1 year	S
With contrast	No	NM	NM	No	NM
**Head MRI**	C	C	C	C	S
Abnormal CT scan	Yes	NM	Yes	Yes	NM
Suspicion of abuse without neurological symptoms	No	NM	Yes	NM	NM

© 2021 Blangis F et al. *JAMA Network Open.*

**eTable 3.** Recommended Imaging and Laboratory Tests for Suspicion of Physical Abuse (continued)					
**Source**	**Nasjonalt kunnskapassenter om vold og traumatisk stress**[12]	**Asociación Española de Pediatría and Sociedad Española de Urgencias Pediátricas**[13]	**Swedish Pediatric Society**[14]	**Swedish Paediatric Radiology Society**[15]	**Swiss Society of Paediatrics**[21]
Sequences	Head: Axial T2, coronal T2, coronal and axial FLAIR, T1 volume series, susceptibility weighted series, diffusion weighted imaging. Contrast-enhanced T1 volume series can be considered. Spinal canal: Sagittal T1, STIR and T2 weighted recordings, possibly axial recordings if visible pathology on the sagittal (axial T1 and T2 over the relevant area).	NM	NM	T1 sagittal and axial<br>T2 axial<br>T2 flair coronal<br>Susceptibility weighted imaging or equivalent<br>/ diffusion	NM
With contrast	Yes (C)	NM	NM	NM	NM
Sedation	Yes	NM	NM	NM	NM
**Spinal MRI**	Yes	NM	C	C	NM
Complete spine	No	NM	Yes	Yes	NM
**Cranial ultrasonography**	NM	C	NM	NM	NM
**EEG**	NM	NM	C	NE	NM
	Detection of thoracic or abdominal injury				
**Chest CT**	C	NM	C	C	NM
With contrast	NM	NM	NM	NM	NM
**Abdominal ultrasonography**	NM	NM	NM	NM	C
**Abdominal CT**	C	NM	C	C	C
With contrast	Yes	NM	NM	NM	NM
**Pelvis CT**	NM	NM	NM	NM	NM
With contrast	NM	NM	NM	NM	NM
**Laboratory tests**					
Liver enzymes	C	C	S	NE	C
Pancreatic enzymes	C	NM	NM	NE	C
Renal function	C	NM	S	NE	C
Urinalysis	C	NM	S	NE	C
Troponin, creatine kinase	C (creatine kinase)	NM	S (troponin)	NE	NM
	Differential diagnosis				
Calcium, phosphorus, alkaline phosphatase	C (+ Na)	NM	S (+ K, Na)	NE	C (phosphorus, alkaline phosphatase: NM, + Na, K, Cl)
25-hydroxyvitamin D, PTH level	C	NM	S	NE	NM
Skin biopsy for fibroblast culture and/or venous blood for DNA analysis for osteogenesis imperfecta	C	NM	NM	NE	NM
CBC with platelets, coagulation regular (PT/INR/aPTT/fibrinogen)	C (PT: NM)C	S (coagulation: U)	S (+lactates)	NE	C (aPTT: NM)
Coagulation advanced (factor VIII level/factor IX level/VWF activity)	C (+ factor VII + vitamin K)	NM	S	NE	C (VWF activity, + factor XIII)
Serum copper, ceruloplasmin and vitamin C levels	C (vitamin C: NM)	NM	S (vitamin C: NM)	NE	NM
Urine organic acids*	C	NM	NM	NE	C
New-born screen review	NM	NM	NM	NE	C

CT, computed tomography; MRI, magnetic resonance imaging; EEG = electroencephalography
PTH, parathyroid hormone; CBC, complete blood cell count; PT, prothrombin time; INR, international normalized ratio; aPTT, activated partial thromboplastin time; VWF, von Willebrand factor ; K, Potassium, Na, Sodium, Cl, Chloride.
S, systematic; NM, not mentioned; C, case-by-case-basis; NE, not expected; U, unclear
[a] Performed for the detection of glutaric aciduria type 1

© 2021 Blangis F et al. *JAMA Network Open.*

**eTable 3.** Recommended Imaging and Laboratory Tests for Suspicion of Physical Abuse (continued)					
**Source**	**The Royal College of Radiologists and The Society & College of Radiographers**[16]	**Royal College of Paediatrics and Child Health**[17]	**American Academy of Pediatrics**[18]	**American College of Radiology**[19]	**American Academy of Pediatrics**[20]
Country	**UK**	**UK**	**USA**	**USA**	**USA**
Year	**2018**	**2019**	**2015**	**2017**	**2020**
Objective for guidance	Physical abuse (imaging tests)	Physical abuse	Physical abuse	Physical abuse (imaging tests)	Abusive head trauma
	Definition of sentinel injuries				
**Type**	NE	Yes	Yes	NE	Yes
**Location**	NE	Yes	Yes	NE	Yes
**Number**	NE	Yes	Yes	NE	NM
**Size**	NE	No	NM	NE	NM
**Pattern**	NE	Yes	Yes	NE	NM
**According to child age and/or if cruising child**	NE	Yes	Yes	NE	NM
	Detection of skeletal or soft tissues injury				
**Radiological skeletal survey**	S <2 years	S <2 years	S <2 years	S <2 years	S <2 years
Number of views	24-25	24-25	24	24	NM
Views	Frontal and lateral views of the skull, frontal view and lateral views of the chest, frontal view of the abdomen and pelvis, lateral views of the spine, frontal view of the arms (or separate humerus and separate forearm), lateral views of the elbows, wrists, frontal views of the hands and wrists, frontal views of the upper and lower limbs (or separate femur or separate tibia/fibula)	Frontal and lateral views of the skull, frontal view and lateral views of the chest, frontal view of the abdomen and pelvis, lateral views of the spine, frontal view of the arms (or separate humerus and separate forearm), lateral views of the elbows, wrists, frontal views of the hands and wrists, frontal views of the upper and lower limbs (or separate femur or separate tibia/fibula)	Frontal and lateral views of the skull, lateral views of the cervical spine and thoracolumbosacral spine, and single frontal views of the long bones, hands, feet, chest, and abdomen, oblique views of the ribs	Frontal and lateral views of the skull, lateral views of the cervical spine and thoracolumbosacral spine, and single frontal views of the long bones, hands, feet, chest, and abdomen, oblique views of the ribs	NM
**Follow-up skeletal survey**	S (11 to 14 days, max 28 days)	S (11 to 14 days)	C (14 to 21 days)	C (14 days)	NM
Number of views	9 minimum	NM	NM	10 minimum	NM
Views	Any abnormal or suspicious areas + frontal and lateral views of the chest, frontal views of the arms, frontal views of the upper and lower limbs	NM	NM	To limit radiation exposure, pelvis, spine, and skull radiographs can be omitted if no injury was initially seen in these regions	NM
**Bone scintigraphy**	No	C	C	C	NM
Sedation	NM	NM	NM	Yes	NM
**Ultrasonography (bones)**	C	NM	NM	NM	NM
**Whole-body MRI**	No	NM	NM	NM	NM
With contrast	NM	NM	NM	NM	NM
Sedation	NM	NM	NM	NM	NM
	Detection of head or spine injury				
**Eye fundus examination**	NE	S	S	NE	U
Number, type, extent, patterns of retinal haemorrhages	NE	Yes	Yes	NE	S
**Head CT**	S	S <1year, C 1 to 2years old	C	S	S (or MRI)
With contrast	NM	NM	Yes	No	NM
**Head MRI**	C	C	C	C	C
Abnormal CT scan	Yes	Yes	Yes	Yes	S (or MRI)

© 2021 Blangis F et al. *JAMA Network Open.*

Suspicion of abuse without neurological symptoms	Yes	NM	Yes	Yes	NM

© 2021 Blangis F et al. *JAMA Network Open.*

**eTable 3.** Recommended Imaging and Laboratory Tests for Suspicion of Physical Abuse (continued)					
**Source**	**The Royal College of Radiologists and The Society & College of Radiographers[16]**	**Royal College of Paediatrics and Child Health[17]**	**American Academy of Pediatrics[18]**	**American College of Radiology[19]**	**American Academy of Pediatrics[20]**
Sequences	T1 sagittal and axial<br>T2 axial<br>Axial or coronal fluid-attenuated inversion recovery (FLAIR)<br>Axial T2 gradient echo or susceptibility-weighted imaging / diffusion weighted imaging	Diffusion weighted imaging	Diffusion-weighted images	Standard sequences + diffusion weighted imaging and susceptibility weighted imaging +/- contrast-enhanced MRI sequences	NM
With contrast	NM	NM	NM	No	NM
Sedation	NM	NM	NM	Yes	NM
**Spinal MRI**	C	C	C	S	C
Complete spine	Yes	Yes	U	Yes	U
**Cranial ultrasonography**	NM	No	C	NM	No
**EEG**	NE	NM	NM	NE	NM
	Detection of thoracic or abdominal injury				
**Chest CT**	C	NM	NM	C	NM
With contrast	NM	NM	NM	C	NM
**Abdominal ultrasonography**	NM	NM	NM	No	NM
**Abdominal CT**	S	C	C	S	NM
With contrast	NM	Yes	Yes	Yes	NM
**Pelvis CT**	NM	NM	NM	S	NM
With contrast	NM	NM	NM	Yes	NM
**Laboratory tests**					
Liver enzymes	NE	C	S	NE	NM
Pancreatic enzymes	NE	C	S	NE	NM
Renal function	NE	NM	NM	NE	NM
Urinalysis	NE	NM	C	NE	NM
Troponin, creatine kinase	NE	NM	S	NE	NM
	Differential diagnosis				
Calcium, phosphorus, alkaline phosphatase	NE	C	S	NE	NM
25-hydroxyvitamin D, PTH level	NE	C	C	NE	NM
Skin biopsy for fibroblast culture and/or venous blood for DNA analysis for osteogenesis imperfecta	NE	C	C	NE	NM
CBC with platelets, coagulation regular (PT/INR/aPTT/fibrinogen)	NE	C	S	NE	NM
Coagulation advanced (factor VIII level/factor IX level/VWF activity)	NE	C (factor IX, d-dimer: NM; + factor XIII)	S (+ D-dimer)	NE	NM
Serum copper, ceruloplasmin and vitamin C levels	NE	U (copper, vitamin C) + vitamin A	C	NE	NM
Urine organic acids*	NE	C	C	NE	NM
New-born screen review	NE	NM	S	NE	NM

CT, computed tomography; MRI, magnetic resonance imaging; EEG = electroencephalography
PTH, parathyroid hormone; CBC, complete blood cell count; PT, prothrombin time; INR, international normalized ratio; aPTT, activated partial thromboplastin time; VWF, von Willebrand factor
S, systematic; NM, not mentioned; C, case-by-case basis; NE, not expected; U, unclear
[a] Performed for the detection of glutaric aciduria type 1

© 2021 Blangis F et al. *JAMA Network Open.*

eTable 4. Guidance Expected Given the Scope of the 20 Guidelines Included in the Systematic Review

			Specific guidance expected				
**Country (year)**	**Title (verbatim +/- translation)**	**Scope (verbatim +/- translation)**	**Sentinel injury**	**Skeletal injury**	**Head and spine injury**	**Thoracic or abdominal injury**	**Differential diagnosis**
**Australia (2014)**[2]	"Suspected child abuse and neglect medical protocol"	"This guideline provides a new standard template for medical staff to record a forensically oriented medical assessment of a child or young person, to enable an informed opinion about the probability that injuries have been caused intentionally."	Expected	Expected	Expected	Expected	Expected
**Australia (2017)**[3]	"Guidelines for protecting children 2015"	"The guidelines for protecting children […] provide information, procedural guidance and resources to support WA health system staff to promote and safeguard the wellbeing of children when they are at risk of or have been harmed through abuse and/or neglect."	Expected	Expected	Expected	Expected	Expected
**Canada (2018)**[4]	"The medical assessment of fractures in suspected child maltreatment: infants and young children with skeletal injury"	-	Expected	Expected	Expected	Expected	Expected
**France (2017)**[5]	"Shaken baby syndrome or non-accidental shaking head injury"	"The objectives are to improve the recognition of the shaken baby syndrome by health professionals and to specify the approach and diagnostic criteria; to specify the possible mechanisms of injury; to specify the action to be taken to protect the infant."	Expected	Expected	Expected	Expected	Expected
**Germany (2019)**[6]	"Child maltreatment, abuse, neglect with the involvement of youth welfare and pedagogy (child protection guideline)"	-	Expected	Expected	Expected	Expected	Expected
**Ireland (2020)**[7]	"Guidelines for investigation of children with suspected physical abuse"	"To provide clinical guidelines for the radiological investigation of children with suspected physical abuse"	Not expected	Expected	Expected (except eye fundus examination)	Expected	Not expected
**Israel (2014)**[8]	"Identification and treatment in minors' victims of abuse and neglect"	"This guideline regulates the modalities of treatment by the health services in the hospital and in the community, in all cases where there is a suspicion of harm and abuse of the juvenile by the person in charge."	Expected	Expected	Expected	Expected	Expected
**Japan (2014)**[9]	"Child abuse treatment guide"	-	Expected	Expected	Expected	Expected	Expected
**Netherland (2016)**[10]	"Guideline on identifying child abuse in emergency medical care"	"The directive focuses on all children presenting in emergency medical care. The described next steps are exclusively for those children who are suspected of child abuse."	Expected	Expected	Expected	Expected	Expected
**New Zealand (2016)**[11]	"Abuse and neglect"	"This guideline is intended to help you in the appropriate assessment and initial management of children and young people where you become concerned about possible abuse or neglect."	Expected	Expected	Expected	Expected	Expected
**Norway (2018)**[12]	"Investigation of children on suspicion of violence and abuse"	-	Expected	Expected	Expected	Expected	Expected
**Spain (2010)**[13]	"Child abuse. Intervention in the emergencies"	-	Expected	Expected	Expected	Expected	Expected
**Sweden (A) (2019)**[14]	"Medical investigation and collaboration in case of suspicion of abuse of young children"	-	Expected	Expected	Expected	Expected	Expected
**Sweden (B) (2019)**[15]	"Guidelines for radiology in the investigation of suspected physical child abuse"	-	Not expected	Expected	Expected (except eye fundus examination)	Expected	Not expected
**Switzerland (2017)**[21]	"Guidelines for child protection activities in paediatric hospitals"	"[...] guidelines for the diagnosis and management of child abuse and neglect."	Expected	Expected	Expected	Expected	Expected
**UK (2018)**[16]	"The radiological investigation of suspected physical abuse in children"	"This guidance is designated to assist referring clinicians, paediatricians, radiologists, radiographers and nuclear medicine technologists who are requesting, performing or reporting on imaging in such cases […]"	Not expected	Expected	Expected (except eye fundus examination)	Expected	Not expected
**UK (2019)**[17]	"Recognition of physical abuse. Good Practice recommendations"	"This chapter provides a guide to the features of childhood injury that should raise suspicion of physical abuse and the assessment and investigations that should be performed to determine the likelihood of physical abuse."	Expected	Expected	Expected	Expected	Expected
**USA (2015)**[18]	"The evaluation of suspected child physical abuse"	-	Expected	Expected	Expected	Expected	Expected
**USA (2017)**[19]	ACR Appropriateness Criteria® Suspected Physical Abuse—Child	"These criteria are intended to guide radiologists, radiation oncologists, and referring physicians in making decisions regarding radiologic imaging and treatment."	Not expected	Expected	Expected (except eye	Expected	Not expected

© 2021 Blangis F et al. *JAMA Network Open.*

					fundus examination)		
**USA (2020)**[20]	Abusive head trauma in infants and children	"This statement aims to provide pediatric practitioners with general guidance on a complex subject."	Expected	Expected	Expected	Expected	Expected

© 2021 Blangis F et al. *JAMA Network Open.*

eTable 5. Expected and Not Expected Statements Given the Scope of Guidelines for the Diagnosis of Child Abuse

**Expected statement**[a]	**Guideline scope**	
	**General guidance including imaging and laboratory tests**	**Guidance for the imaging tests to be performed**
**Radiological skeletal survey**	Expected	Expected
**Follow-up skeletal survey**	Expected	Expected
**Bone scintigraphy**	Expected	Expected
**Eye fundus examination**	Expected	Not expected
**Head CT**	Expected	Expected
**Head MRI**	Expected	Expected
**Spinal MRI**	Expected	Expected
**Cranial ultrasonography**	Expected	Expected
**Chest CT**	Expected	Expected
**Abdominal ultrasonography**	Expected	Expected
**Abdominal CT**	Expected	Expected
**Liver enzymes**	Expected	Not expected
**Pancreatic enzymes**	Expected	Not expected
**Renal function**	Expected	Not expected
**Urinalysis**	Expected	Not expected
**Troponin, creatine kinase**	Expected	Not expected
**Calcium, phosphorus, alkaline phosphatase**	Expected	Not expected
**25-hydroxyvitamin D, PTH**	Expected	Not expected
**Skin biopsy for fibroblast culture and/or venous blood for DNA analysis for osteogenesis imperfecta**	Expected	Not expected
**Serum copper, ceruloplasmin and vitamin C levels**	Expected	Not expected
**Urine organic acids**	Expected	Not expected
**CBC with platelets, coagulation regular (PT/INR/aPTT/fibrinogen)**	Expected	Not expected
**Coagulation advanced (factor VIII level/factor IX level/d-dimer/VWF activity)**	Expected	Not expected

CT, computed tomography; MRI, magnetic resonance imaging; US, ultrasonography
PTH, parathyroid hormone; CBC, complete blood cell count; PT, prothrombin time; INR, international normalized ratio; aPTT, activated partial thromboplastin time; VWF, von Willebrand factor

[a] Suggested in >2 of the 20 included guidelines

eReferences

1. United Nations. Country classification. https://www.un.org/en/development/desa/policy/wesp/wesp_current/2014wesp_country_classification.pdf. Published 2014. Accessed March, 2020.
2. New South Wales Government. Suspected Child Abuse and Neglect (SCAN) medical protocol. https://www1.health.nsw.gov.au/pds/ActivePDSDocuments/GL2014_012.pdf. Published 2014. Accessed July, 2020.
3. Government of Western Australia Department of Health. Guidelines for protecting children. https://ww2.health.wa.gov.au/~/media/Files/Corporate/general%20documents/Child%20protection/PDF/Guidelines-for-protecting-children-2015.ashx. Published 2017. Accessed July, 2020.
4. Canadian Paediatric Society. The medical assessment of fractures in suspected child maltreatment: infants and young children with skeletal injury https://www.cps.ca/en/documents/position/fractures-in-suspected-child-maltreatment. Published 2018. Accessed 07, 2020.
5. Syndrome du bébé secoué ou traumatisme crânien non accidentel par secouement. Évaluation et recommandations Haute Autorité de Santé (HAS) https://www.has-sante.fr/portail/jcms/c_2794425/fr/syndrome-du-bebe-secoue-ou-traumatisme-cranien-nonaccidentel-par-secouement. Published 2017. Updated 2017. Accessed Sept, 2020.
6. Arbeitsgemeinschaft der Wissenschaftlichen Medizinischen Fachgesellschaften. Kindesmisshandlung, -missbrauch, -vernachlässigung unter Einbindung der Jugendhilfe und Pädagogik (Kinderschutzleitlinie). https://www.awmf.org/uploads/tx_szleitlinien/KF_027-069k_Kinderschutz_2018-10.pdf. Published 2019. Accessed July, 2020.
7. Children's Health Ireland Clinical Guidelines Committee. Guidelines for investigation of children with suspected physical abuse. https://www.olchc.ie/Healthcare-Professionals/Clinical-Guidelines/Guidelines-for-Investigation-of-Children-with-Suspected-Physical-Abuse.pdf. Published 2020. Accessed June, 2021.
8. Association of Family Physicians. Child abuse. https://www.wikirefua.org.il/w/index.php?title=התעללות_בילדים_-_Child_abuse&oldid=107321. Published 2014. Accessed July, 2020.
9. Japan Pediatric Society. Child abuse treatment guide. https://www.jpeds.or.jp/modules/guidelines/index.php?content_id=25. Published 2014. Accessed July, 2020.
10. Nederlandse Vereniging voor Kindergeneeskunde. Richtlijn Signalering kindermishandeling in de spoedeisende medische zorg. https://www.nhg.org/sites/default/files/content/nhg_org/uploads/richtlijn_signalering_kindermishandeling_definitief_18_okt_2016.pdf. Published 2016. Accessed.
11. The Paediatric Society of New Zealand. Abuse and neglect. https://www.starship.org.nz/guidelines/abuse-and-neglect/. Published 2016. Accessed July, 2020.
12. Nasjonalt kunnskapassenter om vold og traumatisk stress. Utredning av barn ved mistanke om vold og overgrep. https://voldsveileder.nkvts.no/blog/innhold/vold-mot-barn-utredning/4c-medisinsk-utredning/. Published 2018. Accessed June, 2021.

13. Pou i Fernández J. *Maltrato infantil. Actuación en urgencias. En: SEUP-AEP. Protocolos diagnóstico-terapéuticos de urgencias pediátricas.* 2nd ed: Madrid: Ergon; 2010. p.163-72.
14. Swedish Pediatric Society. Medicinsk rutinhandläggning av misstänkt spädbarnsmisshandel. https://bsfi.barnlakarforeningen.se/wp-content/uploads/sites/13/2019/02/Medicinsk-rutin-f%C3%B6r-handl%C3%A4ggning-av-misst%C3%A4nkt-sp%C3%A4dbarnsmisshandel.pdf. Published 2019. Accessed July, 2020.
15. Swedish Pediatric Radiology Society. Riktlinjer för radiologin vid utredning av misstänkt fysisk barnmisshandel. http://www.sfmr.se/Files.aspx?f_id=165257. Published 2019. Accessed July, 2020.
16. Halstead S, Scott G, Thust S, Hann G. Review of the new RCR guidelines (2017): The radiological investigation of suspected physical abuse in children. *Arch Dis Child Educ Pract Ed.* 2018;104(6):309-312.
17. Royal College of Paediatrics and Child Health. Child protection companion. https://www.rcpch.ac.uk/resources/child-protection-companion-about. Published 2019. Accessed.
18. Christian CW, Committee on Child Abuse And Neglect. The evaluation of suspected child physical abuse. *Pediatrics.* 2015;135(5):e1337-1354.
19. Expert Panel on Pediatric Imaging, Wootton-Gorges SL, Soares BP, et al. ACR Appropriateness Criteria® Suspected physical abuse—child. *J Am Coll Radiol.* 2017;14(5 S):S338-S349.
20. Narang SK, Fingarson A, Lukefahr J. Abusive head trauma in infants and children. *Pediatrics.* 2020;145(4).
21. Swiss Society of Paediatrics. Recommandations pour les activités liées à la protection de l'enfant dans les hôpitaux pédiatriques. http://www.swiss-paediatrics.org/fr/informations/recommandations. Published 2017. Accessed September, 2020.

© 2021 Blangis F et al. *JAMA Network Open.*
