# Supplementary material for: Variations in Guidelines for Diagnosis of Child Physical Abuse in High-Income Countries: A Systematic Review
Source: JAMA Netw Open. 2021 Nov 17;4(11):e2129068. doi: 10.1001/jamanetworkopen.2021.29068 (PMC8600386; doi:10.1001/jamanetworkopen.2021.29068)
Supplement: Supplement 2. — Group Information [file jamanetwopen-e2129068-s002.pdf]

Supplemental Online Content: Nonauthor Collaborators

*Indicates required information. Only first name, last name, and suffix will appear in PubMed.

*Group Name(s): European Confederation of Primary Care Paediatricians (ECPCP) research group							
***First Name and Middle Initial(s)**	***Last Name**	***Suffix (eg, Jr, III)**	Academic Degrees	Institution	Location (city, state/province, country)	Role or Contribution, eg, chair, principal investigator	Group (if more than 1 Group listed in the byline) and/or Subgroup (eg, Steering Committee)
Daniela	Karall		MD, PhD	Clinic for Pediatrics, Division of Inherited Metabolic Disorders, Medical University of Innsbruck	Innsbruck, Austria	Expertise at the national level	
Per	Ashorn		MD, PhD	Department of International Health, School of Medicine, University of Tampere, FI-33521 Tampere, Finland; Department of Paediatrics, Tampere University Hospital	Tampere, Finland	Expertise at the national level	
Andreas	Werner		MD	French Association of Ambulatory Paediatrics	Villeneuve les Avignons, France	Co-author	
Thomas	Fischbach		MD	Professional Association of Pediatricians in Germany	Cologne, Germany	Expertise at the national level	
Jacob	Urkin		MD, MPH	Division of Health in the Community, Faculty of Health Sciences, Ben-Gurion University of the Negev	Beer Sheva, Israel	Expertise at the national level	
Federica	Zanetto		MD	Cultural Association of Paediatricians	Narbolia, Italy	Expertise at the national level	
Patrick	Theisen		MD	Luxembourg Paediatric Society	Esch-sur-Alzette, Luxembourg	Expertise at the national level	
Helena	Porfirio		MD	Portuguese Society of Paediatrics, ambulatory paediatrics section	Coimbra, Portugal	Expertise at the national level	
Concepción	Sanchez-Pina		MD	Spanish Primary Care Paediatric Association	Madrid, Spain	Expertise at the national level	
Paolo	Ramelli		MD	Swiss Paediatric Society	Fribourg, Switzerland	Expertise at the national level	
